# Placental gene signatures associated with high neonatal adiposity: role for immune cell activation

**DOI:** 10.1210/jendso/bvag071

**Published:** 2026-04-02

**Authors:** Jason Laird, Deepak Venkataraman, Hannah Yen, Chelsea Liu, Patrick Catalano, Ruggero Spadafora, Nanguneri Nirmala, Perrie O’Tierney-Ginn

**Affiliations:** Department of Environmental Health and Engineering, Johns Hopkins University, Baltimore, MD 21205, USA; Woman, Mother + Baby Research Institute, Tufts Medicine, Boston, MA 02111, USA; Woman, Mother + Baby Research Institute, Tufts Medicine, Boston, MA 02111, USA; Woman, Mother + Baby Research Institute, Tufts Medicine, Boston, MA 02111, USA; Department of Reproductive Endocrinology, Mass General Brigham, Boston, MA 02115, USA; Woman, Mother + Baby Research Institute, Tufts Medicine, Boston, MA 02111, USA; Department of Pediatrics, Tufts University School of Medicine, Boston, MA 02111, USA; Institute for Clinical Research and Health Policy Studies, Tufts Medicine, Boston, MA 02111, USA; Woman, Mother + Baby Research Institute, Tufts Medicine, Boston, MA 02111, USA; Department of Obstetrics & Gynecology, Tufts University School of Medicine, Boston, MA 02111, USA

**Keywords:** placenta, neonatal adiposity, obesity, microRNA, neutrophil

## Abstract

Fetal fat accumulation is an important indicator of the nutritional environment in pregnancy and placental function. Excessive fat accretion leading to high adiposity at birth, however, can increase a child's long-term risk for obesity and metabolic disease. While maternal body mass index is associated with neonatal adiposity, there is a wide variation in body composition among babies born to women with and without obesity. The placenta orchestrates a complex exchange of nutrients and signals between the mother and baby. To better understand the molecular mechanisms that govern fetal fat accumulation, we profiled the transcriptomics of 79 placentas collected from mothers with and without obesity. We identified a set of 18 neonatal adiposity-associated genes, common to pregnancies with and without obesity. A coexpressed cluster of these genes is involved in innate immune responses, particularly neutrophil activation. We also identified neonatal adiposity-associated genes unique to mothers with or without obesity, suggesting different biological pathways support high newborn adiposity, and/or are responsive to a common initial immune signal. These findings suggest that placental inflammation may influence fetal fat accumulation. Understanding these pathways may help identify novel ways to support healthy fetal growth and reduce the risk of long-term disease.

How babies grow in the womb (organ development, fat accrual, lean tissue growth) can modify their metabolism, cardiovascular function, neurological development, and their risk of future disease [1-4]. Neonatal adiposity is a sensitive marker of the nutritional environment in utero [5], and a difference between 8% and 16% body fat at birth is associated with a 20% greater risk for childhood overweight and obesity [6]. Our group reported that umbilical cord metabolomic profiles are highly associated with neonatal adiposity, but signatures differ depending on maternal obesity status [7]. Maternal obesity (pregravid body mass index [BMI] >30) and insulin resistance are well-recognized risk factors for high neonatal adiposity [8, 9]. However, not all women with obesity have obese babies and the specific maternal and placental factors contributing to fat deposition in utero are poorly understood.

The placenta plays a critical role in mediating the effect of the maternal environment on fetal growth and development. It is a nutritional gatekeeper—sending poorly understood signals to the mother to manipulate her metabolism to free up more nutrients for uptake and transport and modifying fetal delivery via nutrient metabolism and storage. Several studies have reported changes in placental gene expression in response to an obese environment [10-12] but, to our knowledge, the connection between the placental transcriptome and variations in fetal fat accrual has not been reported. In this study, we used untargeted RNA sequencing (RNA-seq) of 79 human placentas from patients with and without obesity, collected at the time of scheduled cesarean delivery to test the hypothesis that there is a placental transcriptomic signature associated with high neonatal adiposity.

## Materials and methods

### Data collection

Data and samples used in this study were collected as part of a prospective cohort of women delivering at term (>37 gestational weeks) via scheduled cesarean delivery from 2004 to 2016. Participants were healthy (absence of high blood pressure, preexisting diabetes, recreational drug or alcohol use, or infectious disease) and without documented fetal anomalies. Maternal demographics, height, and weight were obtained following written informed consent as approved by the institutional review board at MetroHealth Medical Center in Cleveland, Ohio (institutional review board No. 1300650). Net maternal gestational weight gain was calculated as total pregnancy weight gain minus birth weight and placental weight. Maternal fasting blood was collected at time of admission while umbilical cord venous blood was collected at time of delivery. Blood samples were collected into EDTA vacutainers and centrifuged at 2000*g* for 15 minutes to isolate plasma; plasma was aliquoted and stored at −80 °C until analysis. Placental tissue was collected within 30 minutes of delivery, weighed, and sampled immediately. Samples were collected below the placental basal plate, from across the surface, avoiding any macroscopic lesions, and further dissected into small pieces, blotted for blood removal, and snap-frozen in liquid nitrogen. Plasma glucose was measured using a YSI glucose analyzer and insulin via enzyme-linked immunosorbent assay (Millipore catalog No. EZHI-14 K, RRID:AB_2800327) following the manufacturer's instructions. Within 48 hours of birth, neonatal anthropometrics were measured and recorded by a trained research nurse. Birth weight was measured on a calibrated scale, and a measuring board was used for length measurements. The flank skinfold was assessed in the mid-axillary line directly above the iliac crest. Neonatal body composition estimates were made using the following validated equation: fat mass (FM) = 0.39055 (birth weight, kg) + 0.0453 (flank skinfold, mm) – 0.03237 (length, cm) + 0.54657. Lean body mass (LBM) was calculated as birth weight minus fat mass. Percentage body fat (pfat) = fat mass/birth weight × 100 [13].

### Sample selection and grouping

Placental samples were chosen for transcriptomic profiling based on maternal prepregnancy BMI and neonatal adiposity (% body fat). Samples were first categorized by prepregnancy BMI into lean (LE: BMI 18-24.9) or obese (OB: BMI 30-40) groups. Within each BMI category, samples were divided into tertiles by neonatal adiposity based on % body fat at birth. The first (low adiposity, LA) and third (high adiposity, HA) tertiles were chosen for further analysis, creating 4 groups: LE women with low (LELA, N = 20) and high (LEHA, N = 19) adiposity offspring and OB women with low (OBLA, N = 20) and high (OBHA, N = 20) adiposity offspring.

### Placental RNA isolation

We combined and powdered 5 to 6 pieces of frozen tissue representing different areas of each placenta to decrease the effect of variation across the surface. Total placental RNA was extracted by homogenization of approximately 50-mg powdered placental tissue in TRIzol reagent (Invitrogen), following the manufacturer's guidelines. RNA quantification and integrity were measured using an Agilent 2100 Bioanalyzer (Agilent). All samples had an RNA integrity number greater than 7.

### Transcriptomic profiling

Library preparation and sequencing was conducted at Azenta Life Sciences as follows: Library preparation with PolyA selection and Illumina Sequencing RNA samples were quantified using a Qubit 2.0 Fluorometer (Life Technologies) and RNA integrity was checked using an Agilent TapeStation 4200 (Agilent Technologies). RNA-seq libraries were prepared using the NEBNext Ultra II RNA Library Prep for Illumina using the manufacturer's instructions (NEB). Briefly, messenger RNAs (mRNAs) were initially enriched with Oligo d(T) beads. Enriched mRNAs were fragmented for 15 minutes at 94 °C. First-strand and second-strand complementary DNA (cDNA) were subsequently synthesized. cDNA fragments were end repaired and adenylated at 3′ ends, and universal adapters were ligated to cDNA fragments, followed by index addition and library enrichment by polymerase chain reaction (PCR) with limited cycles. The sequencing libraries were validated on the Agilent TapeStation (Agilent Technologies), and quantified by using Qubit 2.0 Fluorometer (Invitrogen) as well as by quantitative (q)PCR (KAPA Biosystems). The sequencing libraries were clustered on the HiSeq (4000 or equivalent) flow cell. After clustering, the flow cell was loaded on the Illumina instrument according to the manufacturer's instructions. The samples were sequenced using a 2 × 150 bp paired end configuration. Image analysis and base calling were conducted by the Control software. Raw sequence data (.bcl files) generated by the sequencer were converted into fastq files and demultiplexed using Illumina's bcl2fastq 2.17 software. One mismatch was allowed for index sequence identification. Six samples were identified as outliers using principal component analysis and removed from further analysis.

### Differential expression analysis and determination of common adiposity associated genes

Placental RNA-seq data were filtered to include only protein coding genes and subsequently split into two groups, neonates with LE and OB mothers. Each group was separately normalized for sequencing depth and dispersion using the R package DESeq2 [14]. Within each maternal group, neonatal adiposity was modeled as a binary factor (LA vs HA) and adjusted for child sex. To assess the robustness of the results, we applied a bootstrap approach. In each group, samples were resampled with replacement 100 times, and differential expression was performed using DESeq2 [14]. Genes with a nominal *P* value below .05 and an absolute fold change (FC) greater than 1.5 were considered differentially expressed genes (DEGs). Genes that were differentially expressed with consistent directionality in 50% or more of the bootstrap iterations were considered further. Multiple hypothesis correction was not applied due to the relatively low statistical power in this analysis. DEGs based on neonatal adiposity in the LE and the OB group were compared to derive a set of “common neonatal adiposity-associated genes” (CNAAGs). The CNAAGs, along with the DEGs unique to the LE and OB maternal groups, underwent Gene Ontology (GO) Enrichment using the R package clusterProfiler [15] to determine the biological context of these DEGs and filtered for terms with a false discovery rate–adjusted *P* value below .1.

### Adiposity-associated genes coexpression analysis

All neonatal adiposity-associated DEGs—either common to both maternal groups or unique within the LE and OB groups—were mapped onto the STRING database (version 12) [16] to determine known protein-protein interactions. Pearson's correlation was then calculated within each maternal group to evaluate whether STRING edges were supported by neonatal coexpression. Edges with an absolute correlation coefficient greater than 0.3 and a *P* value below .05 were considered correlation-confirmed, while other STRING edges were retained for context.

### Tissue specificity of common adiposity-associated genes

The expression of CNAAGs was compared to a placental single-cell RNA-seq (scRNA-seq) reference, collected at term, provided by Pavličev et al in 2017 [17]. In this way, we could determine the typical expression of the common adiposity-associated genes in placental cell types. Given that several CNAAGs were not highly expressed in the placental reference and the strong immune signal observed from the GO enrichment analysis, CNAAG expression was also assessed using the Human Protein Atlas Immune Cell reference [18].

### Placental myeloperoxidase and elastase quantitative polymerase chain reaction

Placenta RNA was extracted as described previously from a subset of the initial cohort (N = 24). cDNA was synthesized using Superscript IV VILO Master Mix (ThermoFisher Scientific) according to the manufacturer’s instructions. The resulting cDNA mix was stored at −20 °C and diluted 10-fold using RNase-free water before use. Gene expression was measured using Applied Biosystems PowerUp SYBR Green Master Mix (ThermoFisher Scientific) in the QuanStudioTM7 Flex Real-Time PCR system (Applied Biosystems). The expression of myeloperoxidase (MPO) (forward 5′-GGTGGGGCTGAGGTACAAAG-3′; reverse—5′-CAGCCCAGCAAGGTCCTAAG-3′) and neutrophil elastase (ELANE) (forward 5′-TCCACGGAATTGCCTCCTTC-3′; reverse 5′-CCTCGGAGGGTTGGATGATA-3′) was analyzed for each placental sample. *RPL17* (forward 5′-TGAACAAAGCACCTAAGATGCGCC-3′; reverse 5′-TGGGCAACCTCCTCTTCTGGTTTA-3′) was used as the housekeeping gene, which had no correlation to maternal or neonatal adiposity. Values were expressed as a ratio of the gene of interest:reference in each sample. The Wilcoxon signed rank test was used to assess differences between adiposity groups. Spearman correlation was used to assess the association between the log2-transformed RNA-seq values vs qPCR validation data for MPO and ELANE.

## Results

Maternal, placental, and neonatal characteristics of the 4 adiposity groups are summarized in Table 1. No differences in maternal age, race, smoking, fasting glucose, gestational age, or neonatal sex were detectable between the groups. Placental weight and neonatal anthropometric measures did not differ between LE and OB women due to group design. Women delivering HA neonates had a higher net weight gain at time of delivery. Prepregnancy BMI did not differ between women delivering LA or HA neonates. HA neonates had heavier placentas, weighed more at birth, were longer, had higher fat mass and fat-free mass, higher cord insulin, and lower cord glucose than LA neonates both in LE and OB mothers. We did not detect a statistically significant interaction between maternal obesity and neonatal adiposity for any of the measured variables.

**Table 1:** Maternal and neonatal characteristics of cohort by maternal body mass index and neonatal adiposity groups

	Maternal BMI <25						Maternal BMI 30-40								
	Low adiposity			High adiposity			Low adiposity			High adiposity					
	Obs	Mean ± SD		Obs	Mean ± SD		Obs	Mean ± SD		Obs	Mean ± SD		*P OB*	*P HA*	P OB*HA
Maternal characteristics															
Age, y	20	27 ± 6		19	29 ± 4		20	29 ± 7		20	27 ± 5		≥.999	≥.999	.12
Pregravid BMI	20	22.5 ± 1.5	A	19	22.1 ± 1.7	A	20	33.9 ± 2.6	B	20	34.7 ± 3.6	B	<.0001	.72	.29
Race, %NHB/%NHW*^a^*	20	45%/55%		19	42%/58%		20	50%/50%		20	45%/55%				
% Smoking*^a^*	20	15%		19	21%		20	15%		20	20%				
Net weight gain, kg	20	11.5 ± 6.8	AB	19	12.9 ± 4.8	AB	20	6.7 ± 7.2	A	20	13.6 ± 9.6	B	.22	.01	.1
Fasting glucose, mg/dL	20	75.2 ± 9.9		19	74.4 ± 6.5		20	76.9 ± 7.8		20	78.8 ± 8.3		.10	.76	.48
Fasting insulin, µU/mL	17	10.5 ± 4.7	A	17	13.6 ± 7.2	A	17	18.1 ± 7.2	B	19	19.8 ± 6.1	B	<.0001	.13	.67
Neonatal characteristics															
Gestational age, wk	20	38.8 ± 0.7		19	39.0 ± 0.7		20	38.9 ± 0.4		20	39.1 ± 0.4		.44	.12	≥.999
Sex, %female*^a^*	20	45%		19	32%		20	60%		20	45%				
Birth weight, kg	20	2.80 ± 0.27	A	19	3.62 ± 0.62	B	20	2.90 ± 0.21	A	20	3.77 ± 0.34	B	.16	<.0001	.78
Birth length, cm	20	48.1 ± 2.0	A	19	49.1 ± 3.5	AB	20	48.6 ± 1.4	AB	20	50.2 ± 1.7	B	.12	.01	.56
Fat mass, kg	20	0.23 ± 0.06	A	19	0.59 ± 0.17	B	20	0.24 ± 0.06	A	20	0.62 ± 0.11	B	.41	<.0001	.68
Fat-free mass, kg	20	2.57 ± 0.23	A	19	3.03 ± 0.47	B	20	2.65 ± 0.16	A	20	3.15 ± 0.24	B	.14	<.0001	.76
% Body fat	20	8.09 ± 1.58	A	19	15.97 ± 2.24	B	20	8.37 ± 1.59	A	20	16.28 ± 1.67	B	.46	<.0001	.97
Untrimmed placental weight, g	20	536.1 ± 123.9	A	19	711. ± 189.7	B	20	559.0 ± 117.0	A	20	787.0 ± 207.9	B	.19	<.0001	.48
Fasting glucose, mg/dL	20	69.0 ± 14.6	A	19	61.1 ± 8.3	B	20	68.3 ± 10.9	AB	18	61.5 ± 12.8	AB	.96	.009	.84
Fasting insulin, µU/mL	17	4.5 ± 2.1	A	17	6.1 ± 3.6	A	19	7.2 ± 3.1	B	18	9.5 ± 5.0	B	.0008	.026	.65

*P* value for effect of maternal obesity (OB); neonatal adiposity (HA); interaction between maternal obesity and neonatal adiposity (OB*HA) calculated by 2-way analysis of variance. Means sharing a letter are not statistically significantly different at the 5% level via Tukey multiple comparison testing.

Abbreviations: BMI, body mass index; NHB, non-Hispanic Black; NHW, non-Hispanic White.

^*a*^Assessed using Fisher exact probability for group differences; race *P* = .99; smoking *P* = .94; neonatal sex *P* = .38.

### Neonatal adiposity-associated genes identified in the placental transcriptome

**Figure 1:**
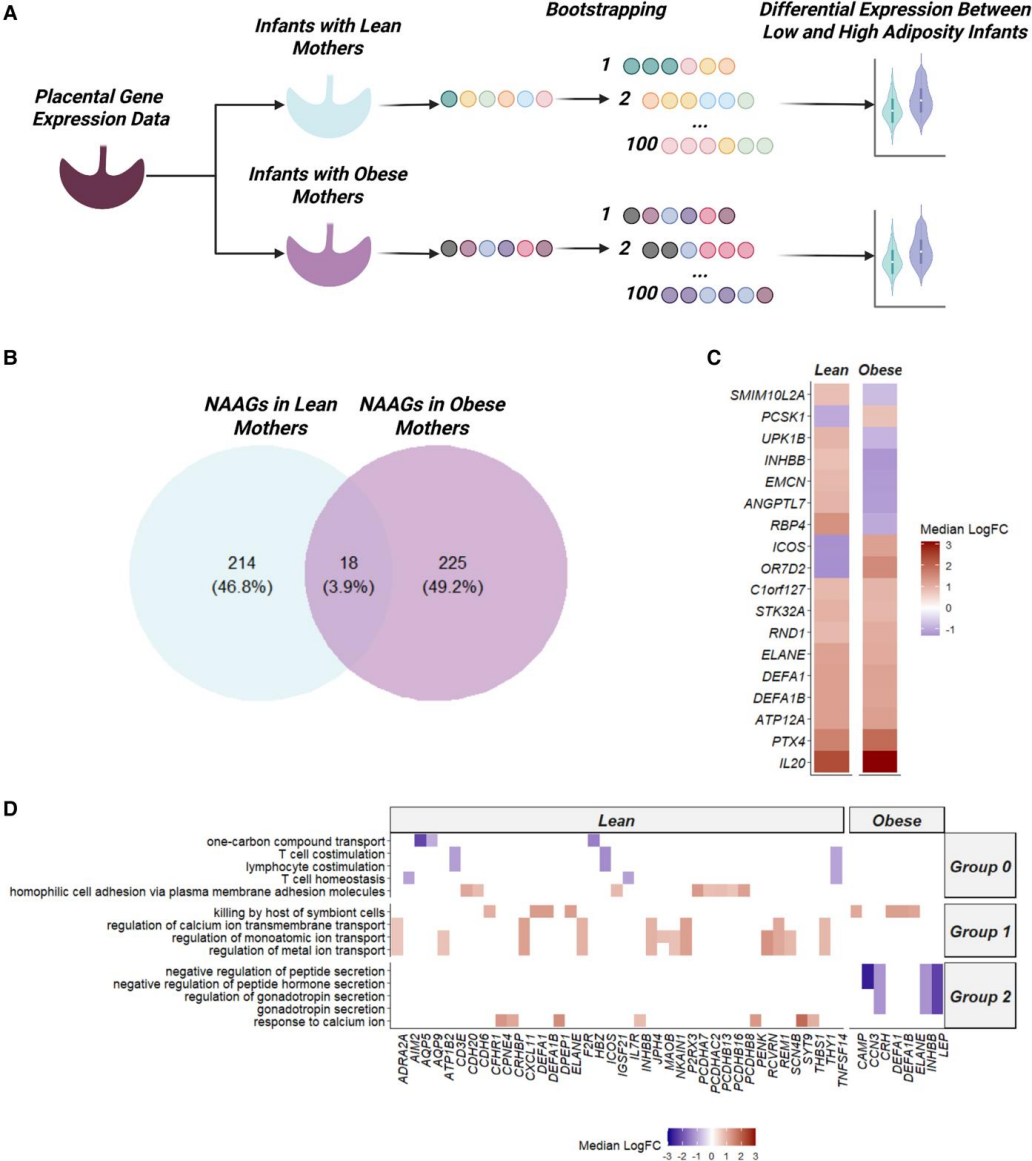
Identification of differentially expressed genes (DEGs) between high- and low-adiposity neonates with lean (LE) and obese (OB) mothers and their associated gene ontology terms. A, Overall differential expression analysis pipeline. B, Overlap between significant DEGs between neonates with LE and OB mothers. Genes were considered statistically significant if the *P* value was below .05 and the absolute value of the fold change was above 1.5. C, Change direction of the common neonatal adiposity-associated genes (CNAAGs) colored by neonate and maternal adiposity status. D, Gene Ontology terms associated with significant DEGs unique to neonates with LE mothers, unique to neonates with OB mothers (NAAGs), and common to neonates with LE and OB mothers (CNAAGs) and colored by the change direction of the DEG (the change direction represents the change in high adiposity neonates). Gene Ontology terms were filtered for terms with a false discovery rate–adjusted *P*-value below .1. Created in https://BioRender.com.

DEGs were determined by splitting the placental RNA-seq data into 2 groups, neonates with LE mothers and those with OB mothers, normalized using DESeq2 [14], and a linear model was constructed using neonatal adiposity status with neonatal sex as a covariate. To improve robustness, samples were bootstrapped, or sampled with replacement, across 100 iterations and genes were retained as differentially expressed if they were nominally statistically significant (*P* < .05; FC > 1.5) in at least 50% of runs with consistent direction of change. Genes meeting these criteria were deemed neonatal adiposity-associated genes (NAAGs). Using this approach, 232 robust NAAGs were identified in the placentas of neonates with LE mothers (Supplementary Table S1) [19] and 243 in neonates with OB mothers (Supplementary Table S2) [19]. The overlap between these 2 sets were used to derive a set of CNAAGs (N = 18) (Table 2; Fig. 1B). Half of the CNAAGs had the same change in direction, while the other half had opposing change in directions between maternal BMI groups (Fig. 1C). Each set of NAAGs, those unique to LE and OB as well as those in common between LE and OB, underwent GO enrichment to determine common biological themes (Supplementary Table S3) [15, 19]. Placentas of HA neonates with LE mothers showed greater expression of genes related to ion transport and reduced expression of T-cell costimulatory genes (Fig. 1D). NAAGs in placentas of neonates with OB mothers were associated with reduced gonadotropin secretion (Fig. 1D). Interestingly, CNAAGs were associated with GO terms related to innate immune response pathways (Fig. 1D).

**Table 2:** Common placental neonatal adiposity-associated genes

		BMI < 25		BMI > 30	
Gene symbol	Gene name	HA vs LA Median Log2FC	Median *P*	HA vs LA Median Log2FC	Median *P*
Genes similarly associated with neonatal adiposity across maternal BMI groups					
*ATP12A*	Potassium-transporting ATPase α chain 2	↑ 1.27	.0017	↑ 1.25	.0031
*C1orf127*	Ciliated left-right organizer ZP-N domains-containing protein	↑ 0.92	.0029	↑ 0.98	.0126
*DEFA1*	Neutrophil defensin 1	↑ 1.25	.0027	↑ 1.21	.0045
*DEFA1B*	Neutrophil defensin 1	↑ 1.26	.0029	↑ 1.2	.0046
*ELANE*	Neutrophil elastase	↑ 1.23	.005	↑ 1.12	.0015
*IL20*	Interleukin-20	↑ 2.38	.0084	↑ 3.13	.0037
*PTX4*	Pentraxin-4	↑ 1.66	.0038	↑ 1.96	.0075
*RND1*	ρ-related GTP-binding protein Rho6	↑ 0.93	.0058	↑ 1.08	.004
*STK32A*	Serine/threonine-protein kinase 32A	↑ 1.03	.0103	↑ 0.96	.0025
Genes differentially associated with neonatal adiposity across maternal BMI groups					
*ANGPTL7*	Angiopoietin-related protein 7	↑ 0.99	.0082	↓ −1.17	.0049
*EMCN*	Endomucin	↑ 0.92	.0099	↓ −1.19	.0045
*ICOS*	Inducible T-cell costimulator	↓ −1.31	.0043	↑ 1.23	.0041
*INHBB*	Inhibin β B chain	↑ 0.84	.0084	↓ −1.25	.0005
*OR7D2*	Olfactory receptor 7D2	↓ −1.32	.005	↑ 1.53	.0076
*PCSK1*	Neuroendocrine convertase 1	↓ −1	.007	↑ 0.82	.0058
*RBP4*	Retinol-binding protein 4	↑ 1.45	.0032	↓ −1	.005
*SMIM10L2A*	Small integral membrane protein 10-like protein 2A	↑ 0.85	.005	↓ −0.79	.0013
*UPK1B*	Uroplakin-1b	↑ 0.98	.003	↓ −0.88	.0021

Abbreviations: BMI, body mass index; FC, fold change; HA, high-adiposity neonate; LA, low-adiposity neonate

### Coexpression of neonatal adiposity-associated genes reveals neutrophil activation signature

**Figure 2:**
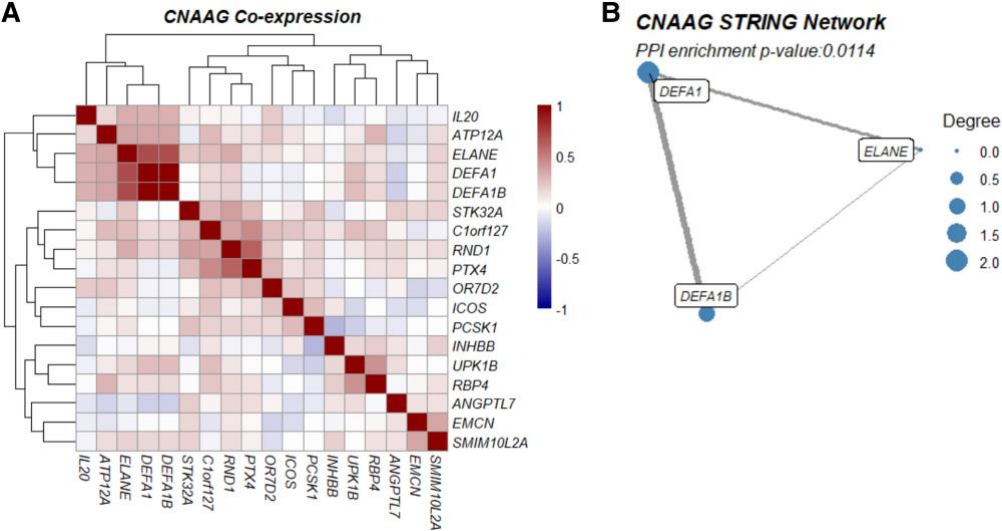
Identification of coexpressing modules among common neonatal adiposity-associated genes (CNAAGs). A, Coexpression of common NAAGs, with modules of interest highlighted. B, STRING Database network (version 12) of CNAAGs, highlighting known interactions between genes.

The STRING network (version 12) [16] of NAAGs among LE mothers revealed a highly connected set of genes (Supplementary Fig. S1A) [19]. The number of predicted protein-protein interactions was statistically significant (*P* = 2.78e-12), and nearly half of these connections (59/120; 49.2%) were coexpressed in placental tissue, where 2 genes were classified as coexpressed if the absolute Pearson correlation coefficient was greater than 0.3 and the *P* value was less than .05. Hub genes, with degree values greater than the 90th degree quantile, included the upregulated genes defensin (*DEF*)*A1*, *DEFAIB*, *ELANE*, *CXCL11*, *MMP1*, *LCN2*, and *F2R*, and downregulated genes *CD3E*, *CXCL3*, *ICOS*, and *IL7R* (see Supplementary Fig. S1A) [19]. The NAAG network among OB mothers was also significantly enriched for protein-protein interactions (*P* = 1.22e-15), with one-third of the STRING connections confirmed by placental coexpression (50/154; 32.5%). Upregulated hub genes included *DEFA1*, *ELANE*, *MPO*, *CAMP*, *CD19*, and *CD1C*, while downregulated hubs included *LEP*, *APOC3*, *FN1*, and *MMP3*. Finally, the STRING network of the 18 CNAAGs was significantly enriched for interactions (*P* = .0114) (Fig. 2B). The only connected components among this network were *ELANE*, *DEFA1*, and *DEFA1B*, which were confirmed by coexpression as well (Fig. 2A). It is notable that *DEFA1* and *ELANE* emerged as consistent hubs across LE, OB, and CNAAG networks.

### Expression of common neonatal adiposity-associated genes in placenta and immune single-cell RNA-sequencing data

**Figure 3:**
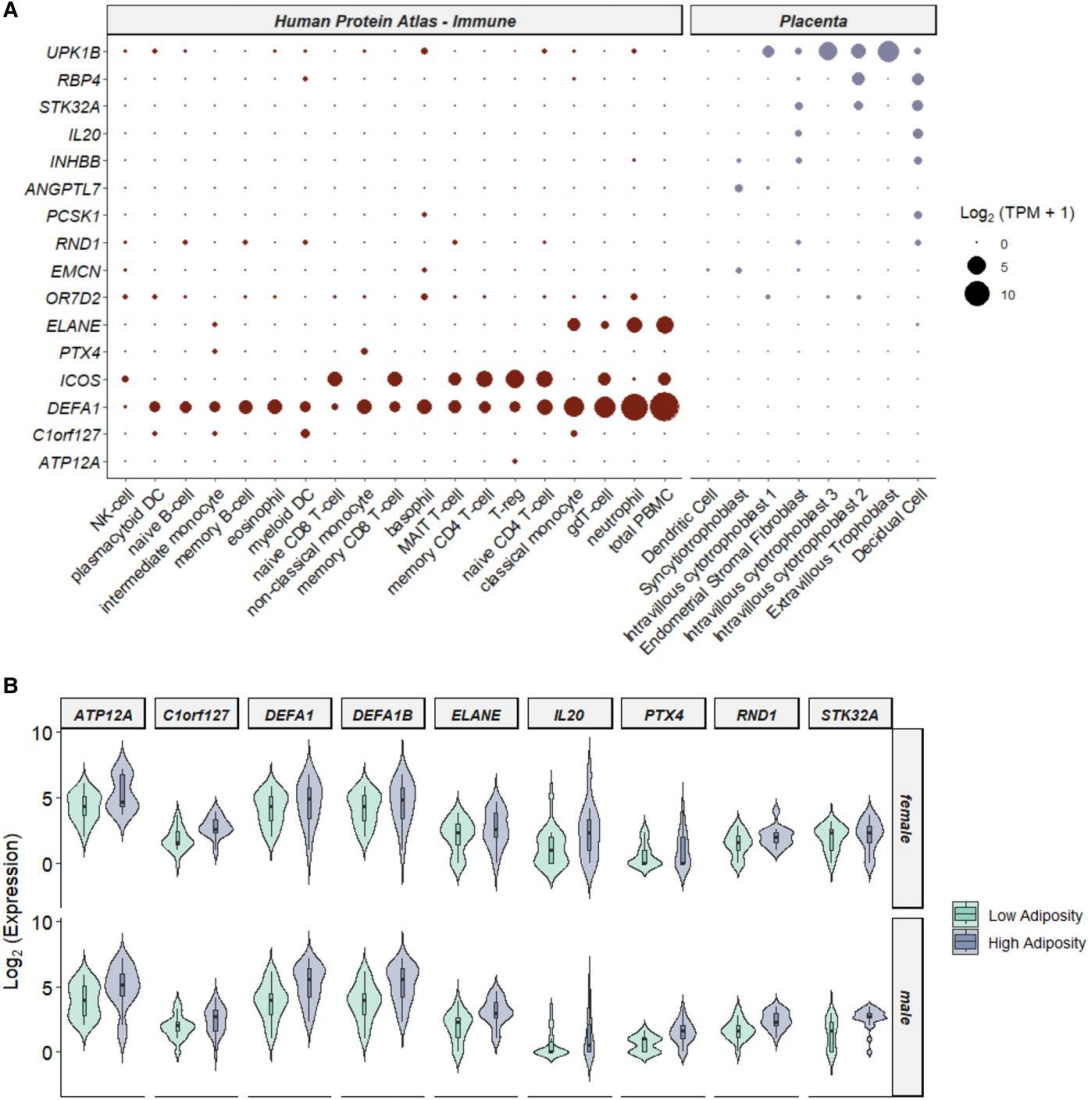
Tissue expression of common adiposity drivers. A, Gene expression of common neonatal adiposity-associated genes (NAAGs) in the Human Protein Atlas immune reference and late-term placenta reference transcriptomic data. B, Log2-transformed gene expression of common NAAGs split by fetal adiposity status and neonatal sex.

The expression of the common adiposity drivers was assessed in 2 scRNA-seq datasets, one placental dataset collected at term [17] and the Human Protein Atlas Immune Cell Web Portal [18]. The placental single-cell dataset was chosen to best represent placental cell composition and gene expression at term, and the Human Protein Atlas Immune Cell data were selected due to the immune signal observed in the GO enrichment of the CNAAGs. Most of the CNAAGs appear to have some native expression in placental tissue collected at term (Fig. 3A). Unexpectedly, a subset of these genes (*ELANE*, *ICOS*, and *DEFA1*) show relatively high expression in T cells, monocytes, and neutrophils, consistent with the GO enrichment results (see Fig. 3A). Common adiposity-associated genes that were not natively expressed in term placenta were mostly well expressed in immune cells (see Fig. 3A) and consistently upregulated in HA neonates (Fig. 3B). Gene expression trends were similar between male and female offspring (see Fig. 3B). This suggests that there may be increased immune cell populations or altered immune cell activation states in the placenta of HA neonates, regardless of maternal adiposity status.

### Placental elastase and myeloperoxidase messenger RNA

**Figure 4:**
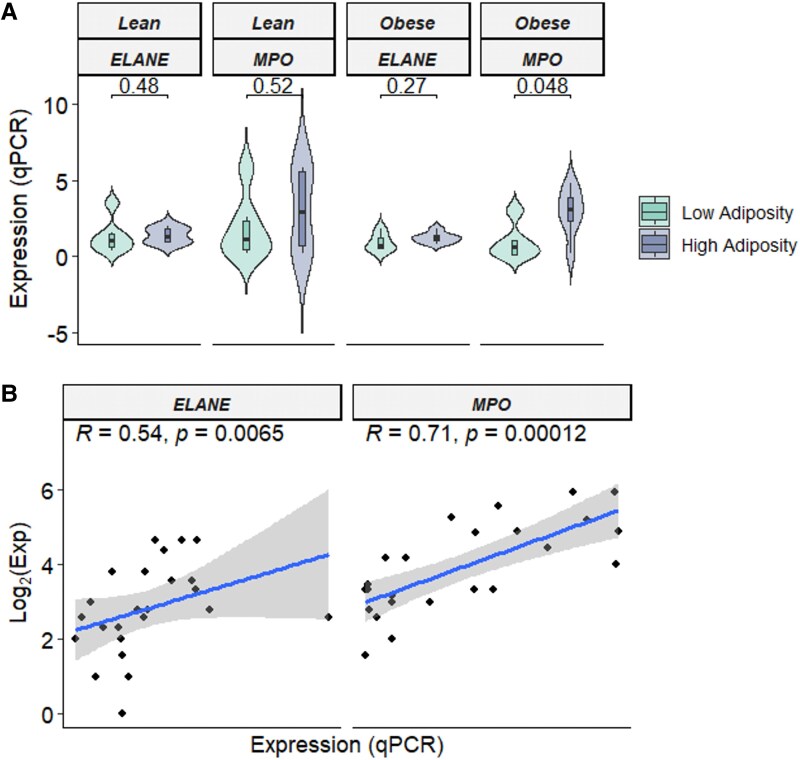
Myeloperoxidase (*MPO*) and elastase (*ELANE*) expression in placenta measured via quantitative polymerase chain reaction (QPCR). A, Placental *MPO* and *ELANE* expression trended higher in the high-adiposity groups, where differences in neonatal adiposity were assessed using the Wilcoxon signed rank test. B, Placental *MPO* and *ELANE* expression measured by QPCR was strongly correlated with their respective log2-transformed RNA sequencing expression, validating our sequencing data. Spearman correlation coefficient (*R*s) and *P* values shown.

We selected a subset of placental samples from each group (N = 6/group) for validation of *ELANE* and *MPO* gene expression. *ELANE* (a CNAAG) and *MPO* (a hub gene in HA offspring of mothers with OB) were selected as they are markers of neutrophil activation—an immune cell type with particularly strong signal shown in Fig 3A. Placental *ELANE* and *MPO* measured by qPCR showed an overall trend to be higher in HA offspring, but only *MPO* in offspring of mothers with OB was statistically significant using a Wilcoxon rank sum test (*P* = .048) in this sample subset (Fig. 4A). Placental *ELANE* (*P* = .0065) and *MPO* (*P* = .00012) measured via qPCR were significantly positively correlated with respective RNA-seq counts (Fig. 4B) when all 24 samples were analyzed together.

## Discussion

A recent study found that a difference in 8% body fat at birth is associated with an increase in 20% of individuals considered overweight/OB at age 5 to 6 years [6]. On average, high maternal BMI is associated with high infant adiposity. However, the correlation between maternal obesity and neonatal adiposity is not universally observed, and other biological variables, likely within the placenta, may be mediating or modifying fetal fat accrual. In this study, we set out to test the hypothesis that there is a placental transcriptomic signature associated with high neonatal adiposity independent of maternal BMI. We identified 18 neonatal adiposity-associated genes common to placentas from LE and OB patients, which revealed that pathways involved in activation of innate immune cells, particularly neutrophils, are associated with high neonatal adiposity in mothers across BMI categories.

Understanding in utero drivers of neonatal adiposity could help identify novel therapeutic targets with the goal of mitigating future morbidities and improving health. We found that in women with and without OB, a common set of 18 genes in the term placenta are associated with neonatal adiposity. Of these, 9 genes are concordantly expressed regardless of maternal BMI—all higher in placentas of HA neonates across all mothers. Several of these elevated genes do not appear to be expressed in the trophoblast, but are expressed in immune cells, particularly neutrophils (*ELANE*, *DEFA1*, *DEFA1B*). Neutrophils are innate immune cells that follow inflammatory cues to migrate to the site of injury during infection or tissue damage [20]. On activation, neutrophils release azurophilic granules that contain *MPO*, *ELANE*, and *DEF1A* [21], all 3 of which we found more highly expressed in placentas of HA infants—though *MPO* was statistically significant only in offspring of OB mothers. There is increasing interest in the effect of neutrophil migration into the placenta [22], which may be important for normal placental development, implantation, and labor initiation. However, excessive infiltration of neutrophils into the placenta may lead to preeclampsia, fetal growth restriction, and recurrent pregnancy loss, suggesting a fine balance must be achieved [22, 23]. Within tissues, *MPO* produces reactive oxidants that are effective against microbial invaders, but when excessive lead to oxidative stress [24]. While the effect of *MPO* on placental function is poorly understood, we and others have shown that placentas of OB patients with HA offspring have impairments in mitochondrial function, greater inflammation, and oxidative stress [11, 25]. Increased expression of all 3 major neutrophil markers—*MPO*, *ELANE*, and *DEFA1—*has been reported in children and adolescents with OB [26-28], supporting a role for neutrophil activation in obesity at a young age. Beyond mere association, these neutrophil markers may be involved in the mechanisms underlying cardiometabolic disease. In a rodent model, *ELANE*-null mice resisted weight gain and neutrophil activation when fed a high-fat diet, while an *ELANE* inhibitor, α1-antitrypsin, administered to high-fat–fed mice reversed weight gain and insulin resistance [29]. Neutrophils are the first cells to infiltrate adipose tissue, following which they activate and recruit other immune cells, initiating an inflammatory milieu characteristic of OB [30]. Additional immune-related genes were higher in placentas of HA offspring of LE and OB mothers, including *IL20*—the gene most strongly elevated in placentas of HA offspring. Interestingly, *IL20* is reported to have anti-inflammatory properties in epithelial cells, targeting and inhibiting inflammatory functions of neutrophils [31]. Conversely, *IL20* levels are elevated in women with OB [32] and this pleiotropic cytokine may play an important role in weight gain and insulin resistance through effects on adipocytes and macrophages [33]. *PTX4* was the second most upregulated placental gene in HA infants. Though its function is poorly understood, it is a member of the pentraxin/pentaxin family of proteins that are involved in acute immunological responses and whose members include C-reactive protein. The pentraxins are involved in the differentiation of monocytes and macrophage function [34], which is consistent with the localization of *PTX4* to monocytes specifically. Together these data suggest that placental inflammation and maternal immune cell activation may play a central role in elevating fetal fat accrual.

Of the 18 placental genes identified to be associated with neonatal adiposity in mothers with and without OB, 9 were discordantly associated between maternal BMI groups. Of the genes that were greater in placentas of HA infants of LE mothers and lower in HA infants of OB mothers (*INHBB*, *UPK1B*, *ANGPTL7*, *RBP4*), a common function was involvement in cellular differentiation and response to stimulus. Genes that were lower in HA infants of LE mothers and higher in HA infants of OB mothers included the immune response gene *ICOS*, which is critical for neutrophil infiltration [35]. *PCSK1*, which is involved in insulin processing, also fits this expression pattern; though its role in the placenta is poorly understood, variants in this gene are associated with OB [36]. It is unclear from these studies how the differential direction of association of these genes with neonatal adiposity among mothers with high or low BMI affects functional outcomes. However, based on our previous report that the association of neonatal adiposity with cord metabolite signatures depends on maternal BMI [7], we speculate that these differences in placental NAAGs between mothers with high or low BMI may underlie the unique metabolic fingerprints of the offspring.

The CNAAGs common to placentas of patients with and without OB were similarly associated with adiposity both among male and female offspring. Though female neonates tend to be smaller at birth, they also tend to have greater adiposity [37]. We have also previously reported that growth outcomes of male neonates are more sensitive to maternal body composition than females [38]. Our present results support a common set of NAAGs in the placenta of male and female offspring independent of maternal OB. Altogether, our results may support a fundamental placental mechanism involving immune cell activation and inflammation underlying at least a subset of HA neonates.

In addition to our 18 CNAAGs, we detected additional NAAGs that were unique to placentas of LE or OB patients. Among placental NAAGs in mothers without obesity, we found that genes related to T-cell costimulation were downregulated and ion transport was upregulated (Fig. 1D). Network analysis of NAAGs in LE mothers identified several downregulated T-cell costimulation genes (*CD3E*, *ICOS*, *IL7R*, *CXCL3*, and *HRNR*) and upregulated innate immune and extracellular regulators (*CXCL11*, *DEFA1/DEFA1B*, *ELANE*, *ENTPD3*, *LCN2*, *MMP1*, *F2R*, *FGF17*, *NES*, and *CD177*) as network hubs (see Supplementary Fig. S1A) [19]. Indeed, T-cell costimulation has been reported to prevent adipose inflammation via regulatory T-cell accumulation [39]. Downregulation of adaptive immune pathways is consistent with the observation that inflammatory mediators are upregulated. Specifically, CXCL chemokines, like the upregulated NAAG, *CXCL11*, have been associated with adipose tissue inflammation [40]. *LCN2* is a known adipokine tied to metabolic stress in pregnancy, during which higher levels have been associated with gestational diabetes [41]. *MMP1* is a matrix metalloproteinase (MMP), a class of proteins responsible for degrading extracellular matrix proteins, and is upregulated in LE mothers with HA neonates [42]. Reduced MMP activity is associated with preeclampsia and intrauterine growth restriction [43]. Beyond tissue remodeling, MMPs can promote neutrophil infiltration [44]. Upregulation of *ELANE* and *DEFA1*/*B* would be consistent with this hypothesis. Additionally, *CD177*, an upregulated NAAG in women without obesity, is a neutrophil-specific glycoprotein responsible for neutrophil adhesion and activation [45]. This suggests that pregnancies in LE mothers resulting in HA offspring skew toward enhanced neutrophil-driven inflammation and extracellular remodeling while suppressing T-cell signaling.

In mothers with OB, placentas from HA infants showed a unique downregulation of gonadotropin secretion pathways (Fig. 1D). Network analysis of OB NAAGs found several downregulated metabolic and extracellular regulators (*LEP*, *APOC3*, *FN1*, *MMP3*, *SLC1A6*, and *CRH*) and upregulated immune markers (*DEFA1*, *ELANE*, *CAMP*, *MPO*, *CD1C*, and *CD19*) as network hubs (Supplementary Fig. S1B) [19]. The triglyceride-modulating functions of APOC3 are well described, as it is an inhibitor of lipoprotein lipase [46]. However, its role in placental lipid metabolism is unexplored. As an inhibitor of LPL, *APOC3* may lower triglyceride lipolysis at the trophoblast surface, which would reduce fatty acid uptake into the placenta. Thus, lower levels of placental *APOC3*, as we observed in HA neonates, may have the effect of increasing LPL activity and placental lipid uptake—an intriguing hypothesis that remains to be tested. Placental leptin (*LEP*) expression was lower in HA offspring of women with OB. Consistent with the known function of many NAAGs, leptin plays an important role in placental growth and immunomodulation, particularly in early pregnancy [47]. Corticotropin-releasing hormone (*CRH*) was lower in offspring of OB mothers and has roles in energy metabolism and OB. Interestingly, low *CRH* levels have been associated with early adiposity and higher levels have been associated with later increases in offspring growth [48]. Unlike in LE mothers, extracellular matrix remodelers, MMP3 and FN1, were downregulated in placentas of OB mothers with HA offspring. This may suggest blunted placental and vascular remodeling, consistent with the observation that patients with OB have an elevated risk of vascular placental lesions [49]. Upregulated NAAGs unique to OB mothers include *CD1C* and *CD19*, both highly expressed in B cells. Elevated placental B-cell numbers have not been reported in neonates with HA. However, CNAAGs feature several neutrophil-related genes and activated neutrophils express factors like *BAFF*, which can promote B-cell survival [50]. Together this may suggest that OB pregnancies with HA infants show a shift toward metabolic reprogramming, placenta remodeling, and neutrophil and B-cell activity.

Our study has several strengths, including prospective enrollment and data collection of fasting patients at the time of scheduled cesarean delivery, which eliminated variations in gene expression and inflammation secondary to labor. Additionally, we had a diverse group of participants, all with uncomplicated normotensive pregnancies, delivering healthy neonates. We had rigorous measures of neonatal adiposity, and placentas were collected in a standardized fashion that prioritized RNA quality. Our study is limited by the low variation between the HA and LA neonates. Because of this our DEGs were not corrected for multiple hypothesis testing. However, the placental inflammation signal observed suggests these findings are not reflective of noise. The role that parental gene polymorphisms associated with OB play in the placental NAAGs we identified cannot be estimated by this dataset as we did not have genetic information on our participants. Because of the nature of our tissue collection protocol, we were not able to perform scRNA-seq, and future studies will be needed to validate the neutrophil-specific signature we observed among our common NAAGs in this cohort. Moreover, the source of neutrophil signal whether maternal or fetal or both remains to be confirmed.

We conclude that potentially, there is a common inflammatory signal originating in maternal immune cells, enriched with metabolic and cell growth pathways within placentas of offspring with HA at birth. Further experiments are needed to determine if maternal immune cell infiltration stimulates placental functional changes that drive neonatal adiposity, or whether immune activation is a response to the placental pathways underlying high fetal fat accrual.

## Data Availability

Original data generated and analyzed during this study are included in this published article or in the data repositories listed in “References” [19]. The gene expression data discussed in this publication have been deposited in NCBI’s Gene Expression Omnibus [51] and are accessible through GEO Series accession number GSE318865 (https://www.ncbi.nlm.nih.gov/geo/query/acc.cgi?acc=GSE318865). The code used in this analysis can be found at https://github.com/BioNomad/neonatal_adiposity.

## Abbreviations

BMI: body mass index
cDNA: complementary DNA
CNAAGs: common neonatal adiposity-associated genes
*CRH*: corticotropin-releasing hormone
*DEF*: defensin
DEGs: differentially expressed genes
ELANE: neutrophil elastase
FC: fold change
GO: Gene Ontology
HA: high adiposity
LA: low adiposity
LE: lean
MMP: matrix metalloproteinase
MPO: myeloperoxidase
mRNA: messenger RNA
NAAGs: neonatal adiposity-associated genes
OB: obese
qPCR: quantitative polymerase chain reaction
RNA-seq: RNA-sequencing
scRNA-seq: single-cell RNA sequencing
